# Turning solitude into strength: Evaluating the use of positive psychotherapy for emotional empowerment among older Indian adults

**DOI:** 10.6026/973206300212635

**Published:** 2025-08-31

**Authors:** Maheswari Narayanasamy, Venkatesh Mathan Kumar vasudevan, Shankar Shanmugam Rajendran, Marudan Anbalagan, Jayalakshmi Lakshmanan, Vijayalakshmi Arumugam, Christina Anthonysamy

**Affiliations:** 1 Department of Psychiatric Nursing, College of Nursing, Madras Medical College, The Tamil Nadu Dr. MGR Medical University, Chennai, India; 2 Department of Mental Health, Madras Medical College, The Tamil Nadu Dr. MGR Medical University, Chennai, India; 3 Department of Child Health Nursing, College of Nursing, Madras Medical College, The Tamil Nadu Dr. MGR Medical University, Chennai, India

**Keywords:** Geriatric, positive psychotherapy, sense of emptiness, emotional distress

## Abstract

The effect of a nurse-led intervention on emotional distress and feelings of emptiness among elderly individuals transitioning to retirement is of interest. Using a quasi-experimental design with 50 participants in each group, results revealed a significant reduction in both emotional distress and emptiness in the experimental group. Scores for emptiness decreased by 22.57% and scores for emotional distress decreased by 25.21%, while the control group showed minimal change. Thus, we show the positive effects of the intervention on improving life satisfaction and reducing psychological discomfort in older adults.

## Background:

It is of greater importance than ever to address stress and mental health issues in the 60-year-old and older population, which is expected to nearly double from 12% to 22% between 2015 and 2050 [1]. About 28% of adults aged 50 to 80 years reported having at least a few days of depression or hopelessness in the preceding two weeks in January 2024, according to Statista. In addition, 44% of respondents reported feeling stressed [2]. Moreover, 44% reported feeling stressed. Emptiness in old age can manifest as a profound sense of loss or lack of purpose. This feeling may stem from various factors, including the loss of significant others or loved ones, diminished physical capabilities, retirement and reduced social interactions [3]. In older adults, emotional distress can show up as despair, anxiety and overall psychological discomfort. Biological, psychological and social factors can all have an impact on mental health problems, which have been repeatedly linked to older persons [4]. Many older persons suffer from psychological anguish, with prevalence rates ranging from 10.7% to 48%, according to research. About 5-10% of older persons suffer from depression, according to studies and anxiety disorders also have a major impact on this demographic [5]. Therefore, it is of interest to study the effectiveness of a nurse-led intervention designed to alleviate emotional distress and feelings of emptiness, aiming to improve overall life satisfaction and psychological well-being in older adults transitioning to retirement.

## Methodology:

The study used a quasi-experimental, non-randomized control group research design, with 50 senior citizens in each of the experimental and control groups. A non-probability convenience selection method was used to select the participants who met the inclusion criteria. Data were collected using the Subjective Emptiness Scale to assess the sense of emptiness and the Perceived Emotional Distress Inventory to assess emotional distress before and after the intervention. Over four weeks, the experimental group received positive psychotherapy techniques such as Positive visualization, three positive things and guided autobiography and meditation practices. The control group continued with routine care. The Institutional Ethics Committee of Chennai's Madras Medical College approved the investigation (No. IEC-MMC/Approval/43112024, Dated: 19.11.2024). Every participant provided written informed consent and complete confidentiality was maintained throughout the study.

## Results:

Most participants in both experimental (54%) and control (56%) groups were males. The majority in both groups belonged to the 60-70 years age group (50% experimental, 52% control).Primary education was most common (42% experimental, 44% control). Most participants followed the Hindu religion (74% in both groups).The majority were married (72% in both groups). Most participant's family income came from their children (58% experimental, 66% control). Urban residence was common (76% in both groups). Most lived in joint families (66% experimental, 76% control). Diabetes mellitus was the most common health condition (40% experimental, 52% control). Most stayed with their children (66% experimental, 76% control). Initial level of sense of emptiness score in experimental group having moderate level (60.00%), high level scores (40.00%). In control group, 70.00% of them are moderate level, 30.00% of them are having high level of score. Accordingly, both experimental group and control group having moderate level of emotional distress (100.00%). In the participants of experimental group having pretest mean sense of emptiness score was 20.76 (74.14%), which decreased to 14.44 (51.57%) post-test, reflecting a 22.57% improvement. In contrast, the control group showed minimal change, with the pretest mean score of 20.48 (73.14%) slightly reducing to 19.94 (71.21%), showing a mere 1.93% reduction. Regarding perceived emotional distress, the experimental group's pretest mean score was 33.10 (73.56%), which decreased to 21.88 (48.62%) post-test, demonstrating a 24.94% improvement. The control group had a pretest mean score of 33.64 (74.76%), which decreased slightly to 32.62 (72.49%) post-test, showing an improvement of only 2.27%. Experimental group are having 22.57% of sense of emptiness reduction score and control group are having 1.93% of sense of emptiness reduction score and Experimental group are having 24.94% of Perceived emotional reduction score and control group are having 2.27% of Perceived emotional distress reduction score. In the study, Figure 1 (see PDF) compares the sense of emptiness scores between the experimental and control groups. The experimental group demonstrated a significant reduction of 22.57%, while the control group showed a minimal reduction of only 1.93%. Similarly, Figure 2 (see PDF) compares the perceived emotional distress scores, revealing a substantial improvement of 24.94% in the experimental group, compared to a modest 2.27% improvement in the control group. These findings underscore the greater impact of the experimental intervention on both sense of emptiness and emotional distress compared to the control group. The experimental group's post-test sense of emptiness score (14.44 ± 2.21) and perceived emotional distress score (19.94 ± 1.93) showed a significant positive fair correlation (r = 0.36, p = 0.01), indicating that higher emotional distress was associated with a greater sense of emptiness. In contrast, the control group showed a weak, non-significant association (r = 0.15, p = 0.36), suggesting that there is little correlation between this group's feelings of emptiness and emotional discomfort (Table 1 - see PDF & 2 - see PDF). Post-intervention findings indicated a correlation between a few demographic factors and sense of emptiness and emotional distress. Participants' ages ranged from 60-70 years and more educated patients are having low emptiness and exhibited emotional distress among Males, aged 60-70 years and pensioner patients are having low stress.

## Discussion:

The findings of this study validate the efficacy of Positive psychotherapy in considerably lessens the emotional pain and emptiness experienced by geriatric, supporting past research demonstrating the positive psychological effects of positive psychotherapy interventions. These findings are noteworthy because they indicate a 60% of participants experienced a moderate level of emptiness, while 40% had a high level in the experimental group, In the control group, 70% reported moderate emptiness and 30% reported high emptiness These results are similar to what was found by Mani *et al.* (2025) [6] carried out descriptive research in Choolai, Chennai. According to the study, loneliness is more common among the elderly, with 41.7% having a moderate level of loneliness and 16.7% reporting a high level. The substantial improvements from the pre-test to the post-test, where emotional distress decreased by 21.88% and a sense of emptiness decreased by 14.44%, demonstrate the potential of positive psychotherapy to develop a better understanding of reflection on past lives as well as to increase present life pleasure. The sense of emptiness score was decreased to 14.44 (51.57%) in the post-test, reflecting a 22.57% improvement. In contrast, the control group showed minimal change, showing a mere 1.93% reduction. Regarding perceived emotional distress, the experimental group decreased to 21.88 in the post-test, demonstrating a 24.94% improvement. The control group, which slightly decreased to 32.62 (72.49%) post-test, showed only a 2.27% improvement. These indicate that the experimental group experienced a significant drop in both sense of emptiness and emotional distress compared to the control group, highlighting the effectiveness of the intervention. Similarly, Tavakoli *et al.* (2020) [7] conducted a quasi-experimental study to examine the effectiveness of positive psychotherapy in enhancing vitality among the elderly. Their findings indicated that positive psychotherapy significantly increased vitality in older adults, suggesting its potential as an effective intervention to boost vitality, which supports the findings of this study. Age and educational attainment showed significant correlations (p=0.05). Patients aged 60-70 years and those with higher education reported lower emptiness scores. Regarding perceived emotional distress, male patients aged 60-70 years and pensioners exhibited lower distress scores. However, in the control group, no significant associations were found between perceived emotional distress scores and demographic variables.

## Conclusion:

The integration of positive psychotherapy (PPT) into geriatric nursing care represents a pivotal shift toward a comprehensive, person-centered model that addresses the physical, emotional and psychological needs of older adults. As aging brings challenges such as loss of independence, chronic illness, bereavement and social isolation, these stressors often contribute to feelings of emptiness, anxiety and depression. Positive psychotherapy, with its focus on strengths, resilience, gratitude and meaning-making, provides an evidence-based framework that significantly benefits the elderly by fostering a sense of purpose and emotional well-being.
